# Inhibition of Angiotensin Converting Enzyme Impairs Anti-staphylococcal Immune Function in a Preclinical Model of Implant Infection

**DOI:** 10.3389/fimmu.2020.01919

**Published:** 2020-09-11

**Authors:** Rishi Trikha, Danielle Greig, Benjamin V. Kelley, Zeinab Mamouei, Troy Sekimura, Nicolas Cevallos, Thomas Olson, Ameen Chaudry, Clara Magyar, Daniel Leisman, Alexandra Stavrakis, Michael R. Yeaman, Nicholas M. Bernthal

**Affiliations:** ^1^Department of Orthopaedic Surgery, University of California, Los Angeles, CA, United States; ^2^Icahn School of Medicine at Mount Sinai, New York, NY, United States; ^3^Divisions of Molecular Medicine and Infectious Diseases, Department of Medicine, Harbor-UCLA Medical Center, Torrance, CA, United States; ^4^The Lundquist Institute for Biomedical Innovation at Harbor-UCLA Medical Center, Torrance, CA, United States

**Keywords:** implant, infection, angiotensin-converting enzyme inhibitor, angiotensin II receptor blocker, bioluminescence

## Abstract

**Background:** Evidence suggests the renin-angiotensin system (RAS) plays key immunomodulatory roles. In particular, angiotensin-converting enzyme (ACE) has been shown to play a role in antimicrobial host defense. ACE inhibitors (ACEi) and angiotensin receptor blockers (ARB) are some of the most commonly prescribed medications, especially in patients undergoing invasive surgery. Thus, the current study assessed the immunomodulatory effect of RAS-modulation in a preclinical model of implant infection.

**Methods:** *In vitro* antimicrobial effects of ACEi and ARBs were first assessed. C57BL/6J mice subsequently received either an ACEi (lisinopril; 16 mg/kg/day), an ARB (losartan; 30 mg/kg/day), or no treatment. Conditioned mice blood was then utilized to quantify respiratory burst function as well as *Staphylococcus aureus* Xen36 burden *ex vivo* in each treatment group. *S. aureus* infectious burden for each treatment group was then assessed *in vivo* using a validated mouse model of implant infection. Real-time quantitation of infectious burden via bioluminescent imaging over the course of 28 days post-procedure was assessed. Host response via monocyte and neutrophil infiltration within paraspinal and spleen tissue was quantified by immunohistochemistry for F4/80 and myeloperoxidase, respectively.

**Results:** Blood from mice treated with an ACEi demonstrated a decreased ability to eradicate bacteria when mixed with Xen36 as significantly higher levels of colony forming units (CFU) and biofilm formation was appreciated *ex vivo* (*p* < 0.05). Mice treated with an ACEi showed a higher infection burden *in vivo* at all times (*p* < 0.05) and significantly higher CFUs of bacteria on both implant and paraspinal tissue at the time of sacrifice (*p* < 0.05 for each comparison). There was also significantly decreased infiltration and respiratory burst function of immune effector cells in the ACEi group (*p* < 0.05).

**Conclusion:** ACEi, but not ARB, treatment resulted in increased *S. aureus* burden and impaired immune response in a preclinical model of implant infection. These results suggest that perioperative ACEi use may represent a previously unappreciated risk factor for surgical site infection. Given the relative interchangeability of ACEi and ARB from a cardiovascular standpoint, this risk factor may be modifiable.

## Introduction

Implant-associated surgical site infections (SSI) represent significant morbidity and mortality for the patient as well as massive economic strain to the current healthcare system (1–7). Despite increasing efforts to prevent SSI through perioperative antibiotic management and the optimization of aseptic surgical technique, infection rates still range from 1.2% in primary joint replacements to 8.6% in ventral hernia mesh repair to as high as 12.9% in ventriculoperitoneal shunts (8–16). Although infection rates and treatment approaches vary by implant type, the overwhelming majority of patients who develop SSI ultimately require surgical implant removal, as bacteria form protective glycocalyx layers on avascular surfaces knows as biofilm (8, 17, 18). In high risk surgeries such as cardiac device implantation and spinal instrumentation, this can lead to catastrophic outcomes such as cardiovascular compromise, spinal column collapse, or death (19, 20). Even in hip and knee replacement surgery, an implant infection carries a worse 5-year mortality rate than breast cancer, renal cell cancer, or HIV/AIDS (21–25). Given the absence of effective treatment for implant infections, prevention is thus paramount. To that end, the identification and optimization of safe and short-term host-targetable risk factors represent crucial, innovative opportunities to prevent SSI.

Angiotensin-converting enzyme inhibitors (ACEi), which block the conversion of angiotensin I to angiotensin II, and angiotensin II receptor blockers (ARB) are two of the most commonly used drugs for the treatment of hypertension (26–29). In 2017, 73 million Americans were prescribed at least one cardiovascular medication, of which, 28 million Americans were prescribed an ACEi and another 15 million were prescribed an ARB (30). Furthermore, according to the CDC, an estimated 11.4% of Americans between 40 and 59 years old, and 21.3% from the age of 60–79 have taken an ACEi in the last 30 days (31). The prevalence of these medications is perhaps even greater in the surgical population. In one multi-institutional study performed across 12 surgery centers, 4,802 out of 14,687 (32.7%) patients who underwent inpatient non-cardiac surgery were taking an ACEi or ARB perioperatively (32).

While the role of the renin-angiotensin system for blood pressure regulation is well-known, emerging evidence suggests this system also has an immunologic function. Of the components involved in this system, ACE appears to have a particularly important role in antimicrobial host defense. Multiple human and animal studies have demonstrated that ACE overexpression increases immune cell response and facilitates host defense against bacterial infections (26, 33–46). In one murine study, selectively reducing ACE expression in neutrophils led to a 6-fold reduction in the clearance of a subcutaneous infection with methicillin-resistant *Staphylococcus aureus* (MRSA) (26). The purported mechanisms underlying any possible immunosuppressive effect of ACE inhibition include dysregulation of TNF-α, IL-6 and/or TGF-β response (47, 48), IL-12 suppression (49), decreased neutrophil superoxide production (26), dysfunctional macrophage activity (34, 35, 40), impaired chemotactic function (34), and decreased pro-inflammatory cytokine production (39, 40). Such mechanisms may impact innate and/or adaptive roles of antimicrobial host defense.

It is crucial to ensure immunocompetency at the time of surgery as the immunoprofile of patients prior to implantation are inextricably linked with the development of a SSI (50–52). It is undoubtedly true that certain patients will not be able to achieve lifelong immunocompetency, however the optimization of the immune system at the time of surgery remains vitally important to minimize SSI, as the majority of implant associated infections occur at the time of surgery (51, 52). Although the immunological impact of ACEi has been studied, to some extent, *in vitro* and in short-term models *in vivo* (33–35, 38, 40–42, 46), there is a lack of longitudinal *in vivo* data to quantify this effect or explore its potential mechanistic basis, and no study to our knowledge has investigated this phenomenon in a surgical model. Thus, the purpose of this study was to assess whether perioperative ACEi treatment impacts the host immune response and determine whether any purported impact would be sufficient to affect infection rates and severity in a well-validated *in vivo* mouse model of implant infection (53–56). This study also aimed to assess whether a reasonable alternative drug with a similar cardiovascular profile could avoid such host immunomodulation, thus optimizing host immunity to minimize perioperative infectious risk.

## Materials and Methods

### Ethics Statement

All animal studies were performed in accordance with protocols reviewed and approved by the Chancellor's Animal Research Committee (ARC) at University of California, Los Angeles (ARC #2012-104-21J). These practices are adherent to National Institute of Health and Public Health Service policies.

### Selection and Preparation of Bioluminescent Xen36 *Staphylococcus aureus*

*Staphylococcus aureus* strain Xen36 (PerkinElmer, Waltham, MA), a bioluminescent strain derived from ATCC-29525 (Wright), was used as the study organism. This strain expresses a genomically integrated luxABCDE operon (53, 57, 58). Consequently, Xen36 generates a bioluminescent blue-green signal with a maximal emission wavelength of 490 nm from viable, metabolically active organisms. Previous studies demonstrated this strain to be ideal for research targeting the longitudinal monitoring of *S. aureus* infections due to its strength and consistency of signal (57–59).

**Figure 1:**
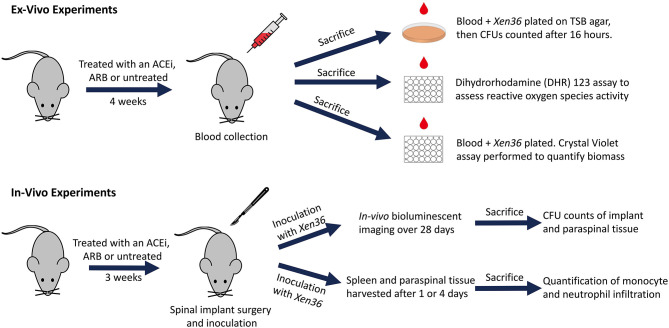
Schematic diagram of *ex vivo* and *in vivo* experiments.

Bacterial inocula were prepared following previously published protocols (53–57). In brief, Xen36 was isolated on kanamycin to select for purity and affirm possession of the kanamycin-resistance marker integral to the lux operon. The authenticated Xen36 strain was then quadrant-streaked onto tryptic soy agar (TSA; Beckton-Dickinson) and incubated for 24 h at 37°C. Single colonies were then isolated and cultured in tryptic soy broth (TSB) for 16 h at 37°C in a shaking incubator (196 rpm) (MaxQ 4,450, Thermo). A subsequent 2 h subculture of a 1:50 dilution of this culture was used to obtain a mid-logarithmic phase bacteria. Lastly, after centrifugation, cells were pelleted, resuspended, and washed in PBS. Bacterial inocula were quantitated and standardized by spectrophotometry (OD, 600 nm; BioMate 3; ThermoFisher Scientific). A schematic overview of our *ex vivo* and *in vivo* experiments is provided (Figure 1).

### *In vitro* Determination of Direct Staphylococcal Growth Effect of ACEi or ARB

In order to confirm that ACEi or ARB do not have any direct antimicrobial effects against *S. aureus*, a Kirby Bauer diffusion susceptibility test was performed (60). Briefly, 20 μL of ACEi, ARB, vancomycin, each at a concentration of 0.5 mg/mL, or normal saline was aliquoted onto separate 6 mm filter paper disks and left to dry for 30 min. One disk from each group was then placed into a separate quadrant of a TSA plate that had been flooded and spread with 200 μL of 1 × 10^6^ *S. aureus* Xen36. This was performed for four total plates. This procedure was replicated on four additional plates using a concentration of 1 mg/mL of study therapeutics. Plates were left to incubate at 37°C for 16 h and zone of inhibition(s) were then analyzed.

### Mice and Medication Administration

Eight to twelve-week-old, 20–25 g C57BL/6 wildtype mice (Jackson Laboratories, Bar Harbor, ME) were housed (four mice per standard cage) and stored with a 12 h light and dark cycle with food and water ad libitum. Veterinary staff assessed all mice on a daily basis to ensure well-being throughout the entirety of the experiment.

Mice were randomized to receive treatment with either: an ACEi (lisinopril; 16 mg/kg/day PO; LKT Laboratories, St. Paul, MN) (ACEi group), an ARB (losartan; 30 mg/kg/day PO, LKT Laboratories, St. Paul, MN) (ARB group), or no treatment (control group) with dosing as per prior independent protocols that demonstrated a percent reduction in blood pressure akin to that of humans (40, 61). Medications were suspended in 250 mL containers of drinking water. Ten milliliters of sucralose was added to the drinking water of all mice and intake was recorded daily to ensure each mouse drank 3–5 mL/day. For all *ex vivo* experiments, mice received medication treatment for 4 weeks prior to cardiac puncture and sacrifice. For *in vivo* experiments, treatment began three weeks preoperatively and continued postoperatively for 4 weeks until sacrifice. These time points were selected based on previous studies demonstrating sufficiently altered immune profiles of mice blood after 7–10 days (26, 35).

### *Ex vivo* Quantification of Respiratory Burst

Following 4 weeks of medication treatment, blood was collected from six mice in each group via cardiac puncture under 2% isoflurane inhalation anesthesia, followed by immediate euthanasia. Ethylenediaminetetraacetic acid (EDTA) was added to blood samples in a 1:10 ratio to prevent coagulation. One-hundred microliters were added from each mouse to each well within a 96-well flat bottom plate (Corning Costar, Corning, New York). Reactive oxygen species (ROS) activity of whole blood were assessed using a dihydrorhodamine (DHR) 123 assay. Briefly, 10 μL of DHR 123 Assay Reagent followed by 25 μL of Phorbol myristate acetate followed by 2 mL of Red Blood Cell Lysis Buffer was added to each plate. Mean fluorescent intensity was read with an excitation filter of 485 nm and an emission filter of 520 nm using a fluorescent plate reader (FLUOstar Omega, BMG Labtech, Ortenberg, Germany).

### *Ex vivo* CFU Quantification of *S. aureus* Mixed With Whole Blood

Six mice in each group underwent whole blood collection as above. Ten microliters of blood from each mouse were then gently mixed with 10 μL of 1 × 10^3^ *S. aureus* Xen36 and incubated at 37°C for 1 h. After 1 h, the entire 20 μL of solution was quantitatively cultured on TSA and incubated at 37°C for 16 h. Resulting CFUs were then counted for a minimum of *n* = 6 replicates in each group.

### *Ex vivo* Quantification of the Biofilm Biomass

Five mice in each group underwent whole blood collection as above. One-hundred microliters of whole blood from each mouse were mixed with 100 μL of 1 × 10^7^ *S. aureus* Xen36 CFU/mL for a final inoculum of 10^6^ CFU in 200 μL. This solution was added to each well within a 96-well-flat bottom plate. Six additional control wells containing 200 μL of saline were also included for standardization. After 24 h of incubation at 37°C, each well was washed with PBS three times to remove residual blood cells and non-adherent bacteria. A well-validated crystal violet assay (Abcam, Cambridge, United Kingdom) (62–64) was performed to quantify the biomass of the residual biofilm formation by OD at 595 nm.

### *In vivo* and Longitudinal Monitoring of Bacterial Burden and Implant and Paraspinal Tissue CFU Quantification

**Figure 2:**
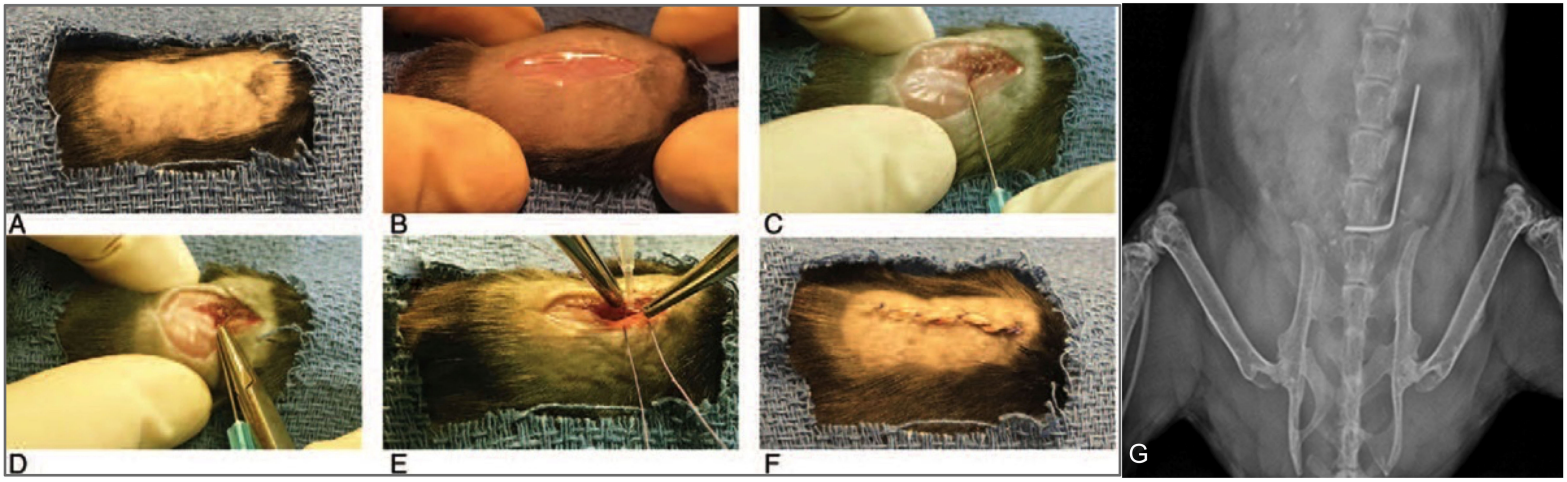
Mouse spinal implant surgery procedures. **(A)** Mice were prepped with six alternating scrubs of povidone-iodine and alcohol and subsequently draped in a sterile fashion. **(B)** A 2 cm midline dorsal incision was made. Dissection was carried through the fascia and muscle and directed laterally along the L4 spinous process. **(C)** The L4 spinous process was reamed with a 25-gauge needle. **(D)** The short arm of the implant was press-fit into the spinous process and the long arm was laid longitudinally parallel along the spine directed cranially. **(E)** The wound was then prepared for closure using polyglycolic acid 5-0 sutures. Prior to these sutures being tied, 1 × 10^2^ CFUs of Xen36 in a volume of 2 μL was inoculated directly onto the long arm of the implant. **(F)** Deep sutures were then tied and a running 5-0 vicryl suture was used to close the skin. **(G)** Proper placement of the implant was confirmed with high resolution X-rays on post-operative day 0 using the IVIS Lumina X5 (PerkinElmer, Waltham, MA).

Twenty-six total mice were randomized into the following groups: 2 in the sterile control group, 8 in the infected control group, 8 in the ACEi group and 8 in the ARB group. Mouse spinal implant surgery and inoculation with 1 × 10^2^ *S. aureus* Xen36 was performed as described in prior protocols (53–56). Briefly, a midline dorsal incision was made and dissection was performed through the fascia and muscle directed laterally along the L4 spinous process. The L4 process was manually reamed with a 25-gauge needle. An “L-shaped” surgical grade 0.1 mm diameter titanium implant (Custom Wire Technologies, Port Washington, WI) was then press-fit into the L4 process. The long arm of the implant measured 6.5 mm in length and the short arm measured 3.5 mm in length (Figure 2). The IVIS Lumina X5 (PerkinElmer, Waltham, MA) was used to capture bioluminescent images representative of *S. aureus* Xen36 burden on postoperative day (POD) 0, 1, 3, 5, 7, 10, 14, 18, 21, 25, and 28. Bioluminescent quantification of bacterial burden was confirmed directly by CFU quantification of the implant and surrounding tissue. On POD 28, each mouse was sacrificed and CFU of bacteria adherent to the implant as well as in the paraspinal tissue quantified. To do so, the implant was sonicated in 500 μL 0.3% Tween-80 (ThermoFisher Scientific) in TSB and the paraspinal tissue was homogenized (Pro200H Series homogenizer; Pro Scientific). Samples from implants and paraspinal tissue were then plated onto a TSA plate and incubated overnight. Resulting CFUs per plate were counted and total CFUs harvested from the paraspinal tissue and implant were expressed as CFUs/mL.

### Histologic Analysis and Quantification of Monocyte and Neutrophil Infiltration

An additional 12 mice were randomized into the following groups: four in the infected control group, four in the ARB group and four in the ACEi group. Mice underwent spinal implant surgery and infection as described above. Two mice in each group were sacrificed on POD1 and two mice in each group were sacrificed at POD4. At the time of sacrifice, paraspinal and splenic tissue samples were harvested and stained with hematoxylin and eosin (H&E), F4/80 antibody (representing monocyte infiltration), and myeloperoxidase (MPO) (representing neutrophil infiltration). Histologic images were de-identified and qualitatively reviewed by a board-certified pathologist to assess for F4/80 and MPO signals. Brightfield slides were digitized on a ScanScope AT (Leica Biosystems, Inc., Vista, CA) and morphometric analysis performed with *Definiens* Tissue Studio (Definiens Inc., Parsippany, NJ) to quantify monocyte and neutrophil counts. Briefly, a stain specific algorithm was created using the pre-defined cellular detection module and classification tool, through which positive and negative stained cells within a tissue core were identified. The data were exported to Excel for further statistical analysis.

### Statistical Analysis

Probability (*p*) values were calculated using a Student's *t*-test (one or two-tailed where indicated), while data analysis among three or more groups were compared using a one-way ANOVA. Longitudinal bioluminescent data were analyzed using a linear mixed effects regression model. Data were expressed as mean ± standard error of the mean (SEM). Stata-14 software (Statacorp, College Station, TX) was used for all statistical analyses and statistical significance was set at *p* < 0.05.

## Results

### *In vitro* Determination of Direct Staphylococcal Growth Effect of ACEi or ARB

**Figure 3:**
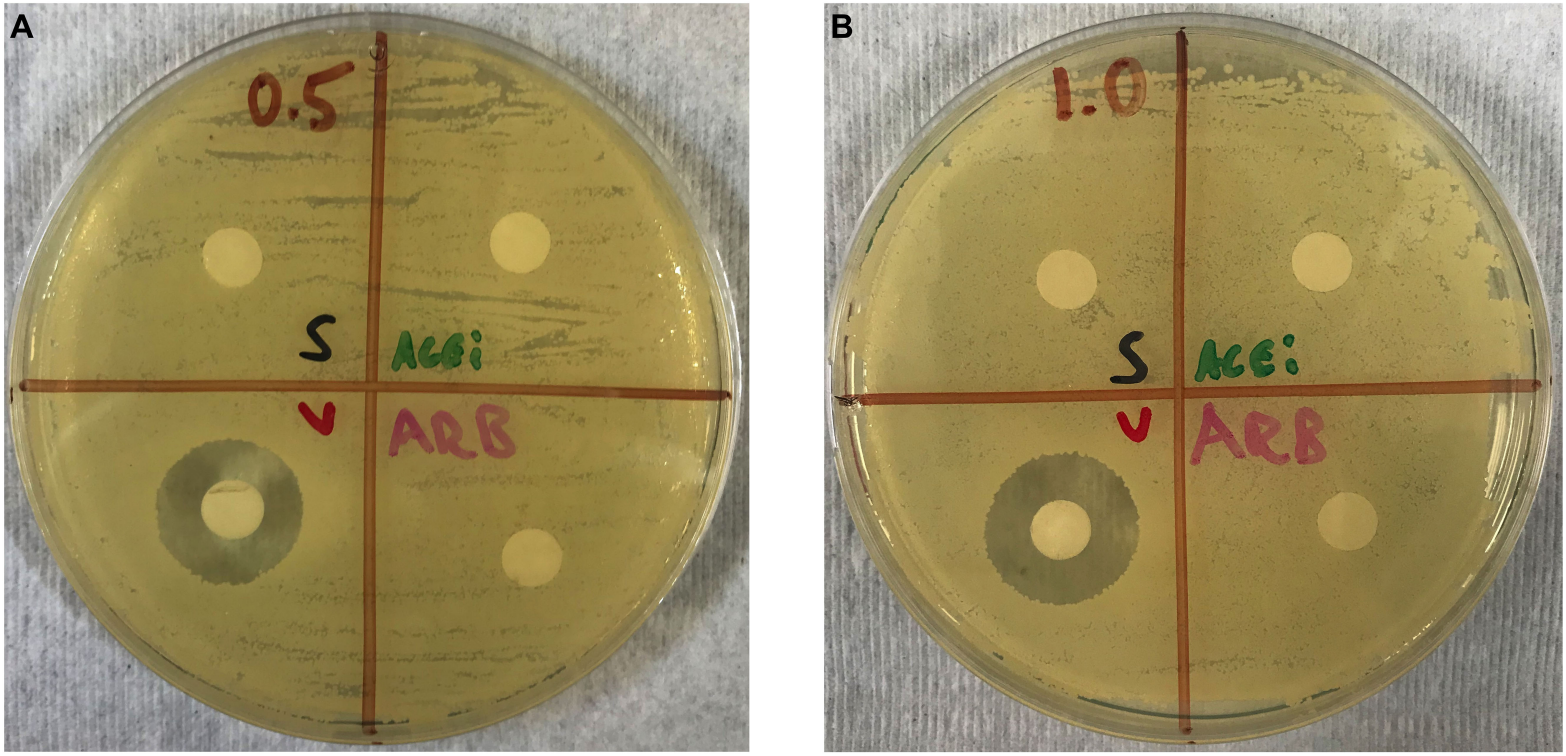
Kirby–Bauer disk diffusion susceptibility test showing no direct effect of ACEi or ARB on *S. aureus* growth when study therapeutics are dosed at 0.5 mg/mL **(A)** and 1.0 mg/mL **(B)**. Thus, any purported effect on bacterial burden in ACEi and ARB-treated mice were not due to any anti-staphylococcal properties of the therapeutics themselves.

No zones of inhibition were appreciated in any plates for the normal saline, ACEi or ARB disks at any concentrations. A zone of inhibition of 18.5 ± 0.3 mm was measured around disks with 0.5 mg/mL of vancomycin. A zone of inhibition of 20.5 ± 0.3 mm was measured around disks with 1.0 mg/mL of vancomycin (Figures 3A,B).

### *Ex vivo* Quantification of Respiratory Burst, CFU, and Biofilm Biomass

**Figure 4:**
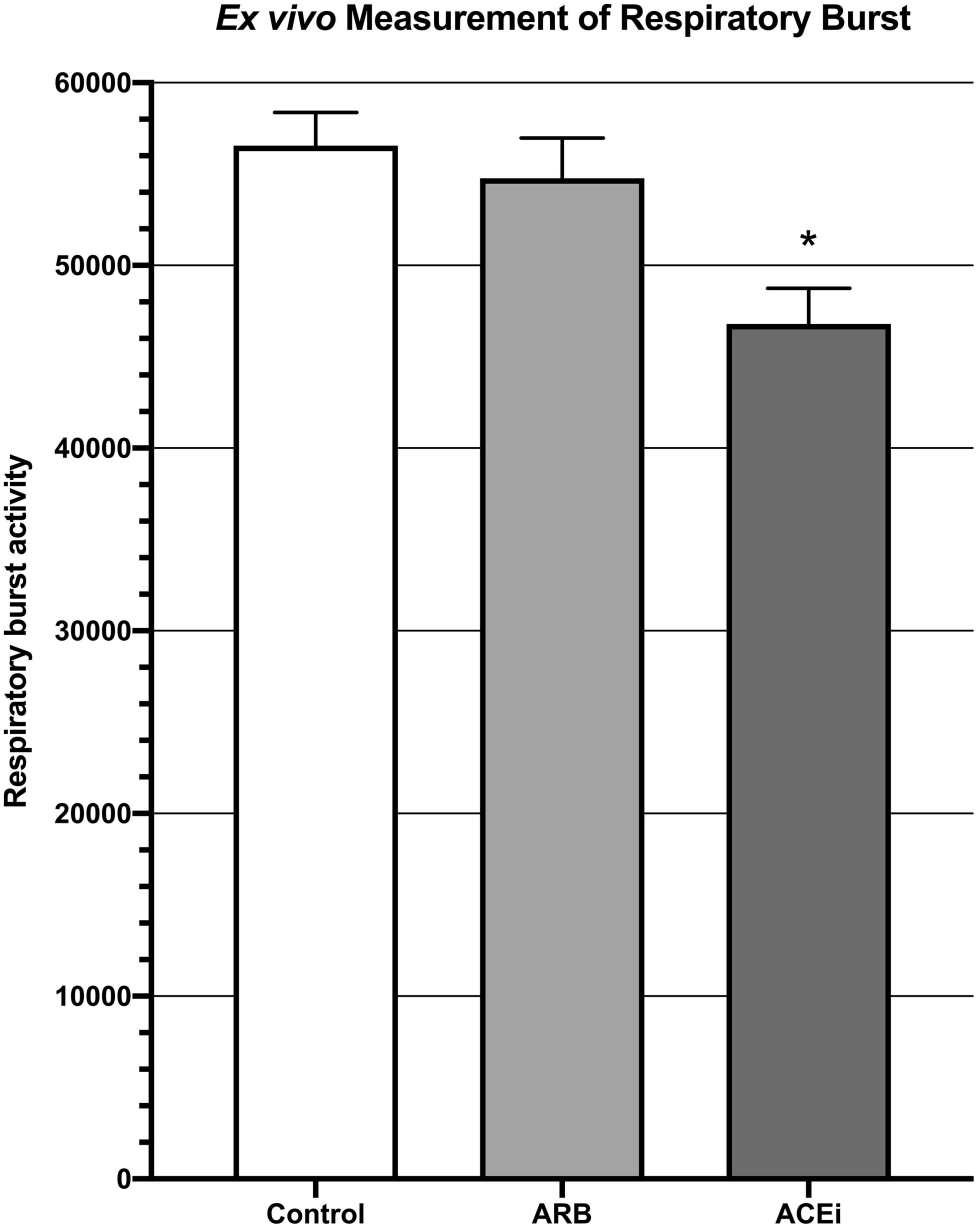
Measurement of respiratory burst *ex vivo* showing significantly decreased respiratory burst in the blood of mice treated with an ACEi as compared to those treated with an ARB and the untreated control group. ^*^p < 0.05.

Respiratory burst, represented by mean fluorescent intensity from a DHR 123 assay, was significantly higher in the blood of mice from the ARB group (5.5 × 10^4^ ± 2.2 × 10^3^) and the infected control group (5.7 × 10^4^ ± 1.8 × 10^3^) when compared to the ACEi group (4.7 × 10^4^ ± 1.9 × 10^3^, *p* < 0.05; Figure 4).

**Figure 5:**
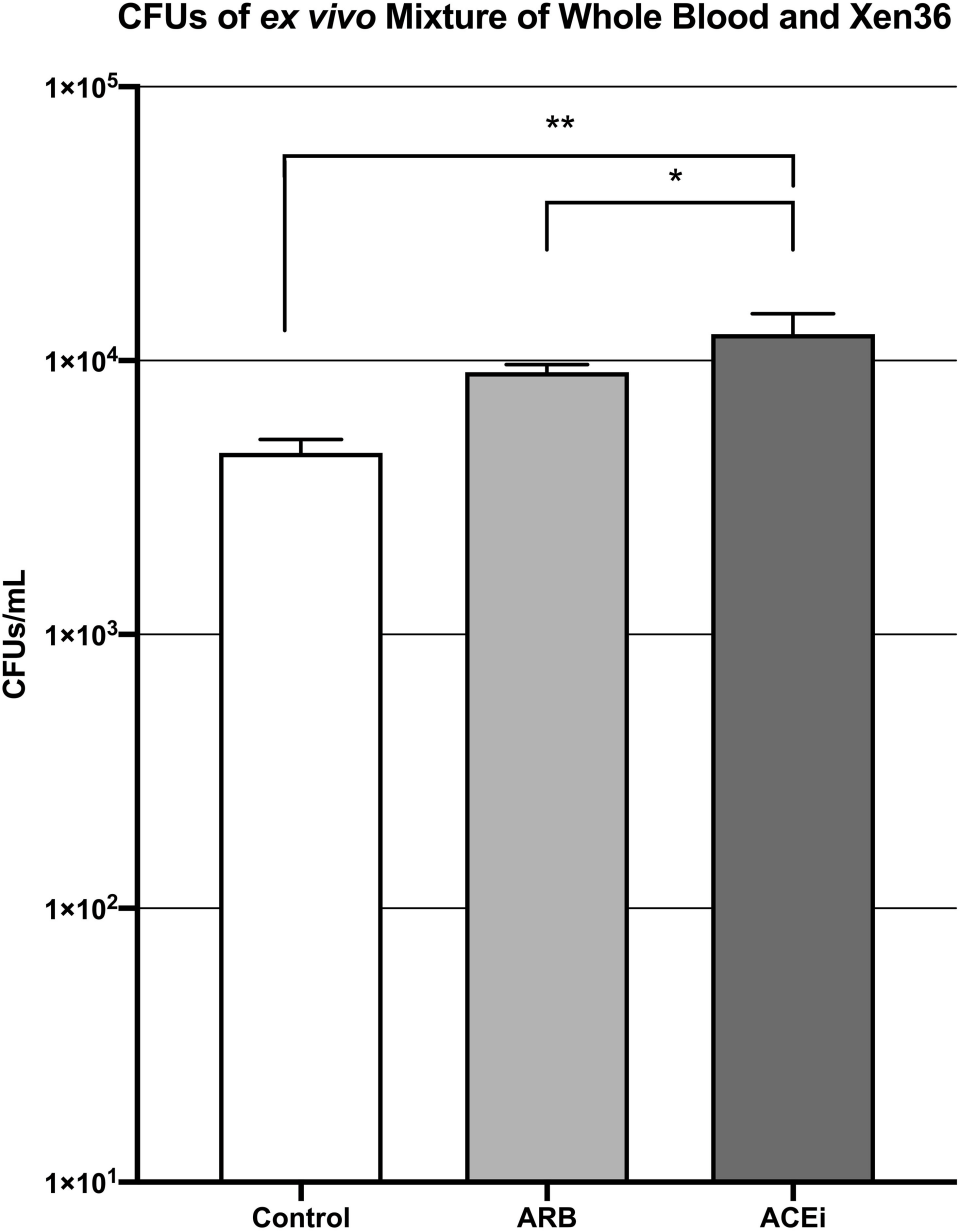
CFU counts demonstrating significantly increased *S. aureus* burden *ex vivo* in the blood of mice treated with an ACEi as compared to those treated with an ARB and the untreated control group. ^*^ denotes *p* < 0.05, ^**^ denotes *p* < 0.01.

The mean CFU/mL of *S. aureus* Xen36 in whole blood was significantly higher for the ACEi group than the ARB group (1.3 × 10^4^ ± 9.5 × 10^2^ vs. 9.1 × 10^3^ ± 2.5 × 10^2^, *p* < 0.05), which was significantly higher than the infected control group (4.6 × 10^3^ ± 2.2 × 10^2^, *p* < 0.05; Figure 5).

**Figure 6:**
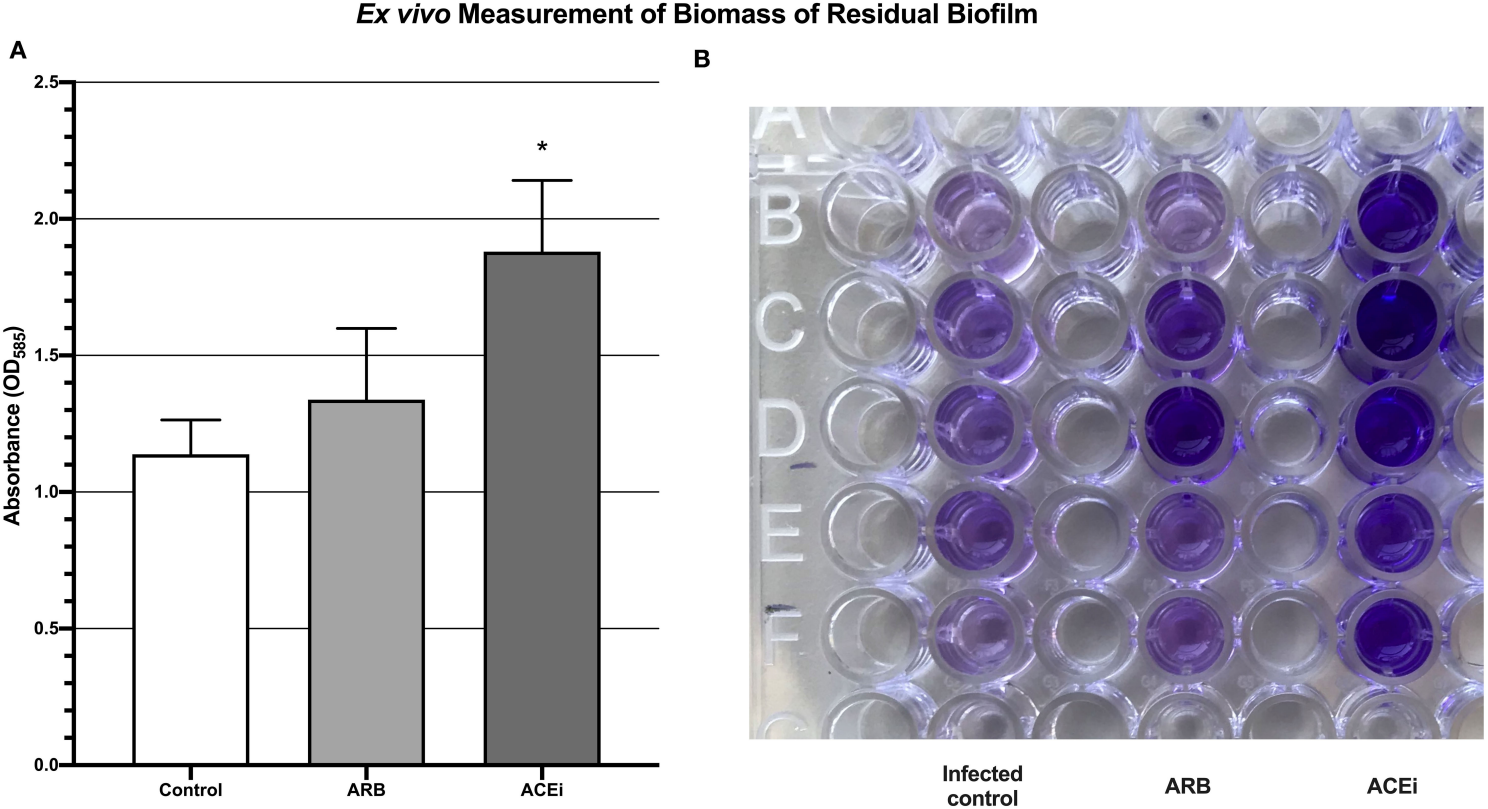
Quantification of biomass of residual biofilm demonstrating a significantly increased *S. aureus* burden *ex vivo* in the blood of mice treated with an ACEi as compared to those treated with an ARB and the untreated group **(A)**. ^*^p < 0.05. Ninety-six well-plate after 24 h of incubation with whole blood and Xen36 for 24 h and stained with crystal violet **(B)**.

Biofilm biomass, represented by absorbance units, was significantly higher in the ACEi group (1.9 ± 0.3) as compared to both the ARB (1.3 ± 0.3) and control (1.1 ± 0.1) groups (*p* < 0.05; Figure 6).

### *In vivo* and Longitudinal Monitoring of Bacterial Burden and Implant and Paraspinal CFU Quantification

**Figure 7:**
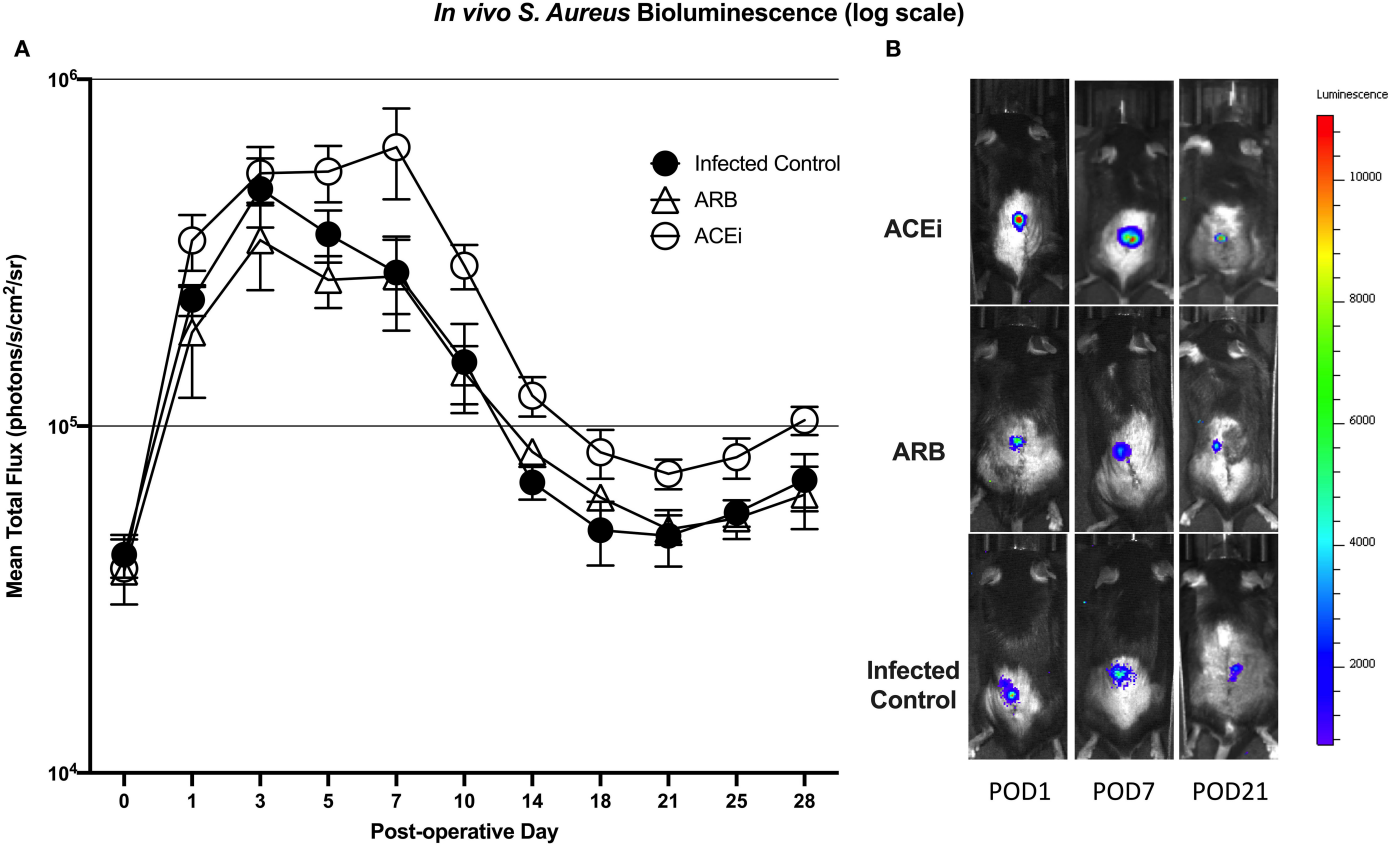
*S. aureus* burden *in vivo* was higher at all time points in mice treated with an ACEi as compared to those treated with an ARB and the untreated group. Statistical significance (*p* < 0.05) was reached at all time points other than POD 0 and 3 **(A)**. Representative Xen36 *S. aureus* bioluminescent images at three selected postoperative time points overlaid on top of grayscale images of mice **(B)**.

Bioluminescent signal was higher in the ACEi group than both the ARB and infected control groups at all time points. This difference reached significance at all time points other than POD 0 and 3 (*p* < 0.05). The only significant difference between the ARB and infected control group occurred at POD 14, when the ARB group was significantly higher than the infected control (Figures 7A,B).

**Figure 8:**
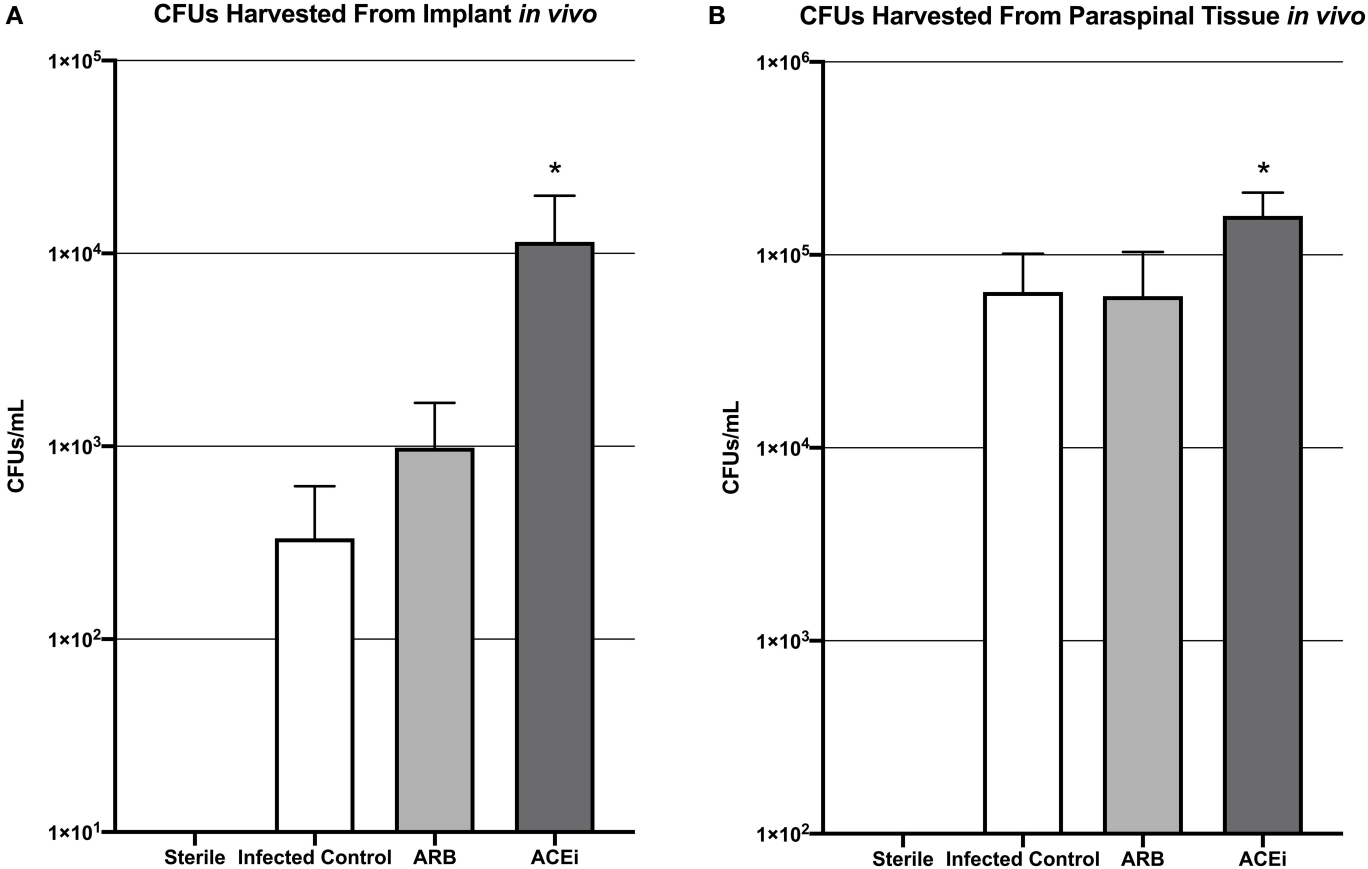
Bacteria harvested from both the implant **(A)** and the paraspinal soft tissue **(B)** demonstrate a significantly increased *S. aureus* burden in mice treated with an ACEi than those with an ARB and untreated mice. ^*^p < 0.05.

Viable *S. aureus* CFUs were identified in 0 of 2 (0%) implants from the sterile control group, 2 of 8 (25%) implants from the infected control group, 2 of 8 (25%) implants from the ARB group, and 3 of 8 (37.5%) implants from the ACEi group. The mean CFU/mL cultured from the harvested implant in the ACEi group (1.1 × 10^4^ ± 8.5 × 10^3^) was significantly higher than either the ARB (9.8 × 10^2^ ± 7.0 × 10^2^) or infected control groups (3.3 × 10^2^ ± 2.9 × 10^2^, *p* < 0.05; Figure 8A).

The mean CFU/mL cultured from the excised paraspinal tissue in the ACEi group (1.6 × 10^6^ ± 5.1 × 10^5^) was significantly higher than the ARB (6.1 × 10^5^ ± 4.2 × 10^5^) and infected control groups (6.4 × 10^5^ ± 3.7 × 10^5^, *p* < 0.05; Figure 8B).

### Histologic Analysis and Quantification of Monocyte and Neutrophil Infiltration

**Figure 9:**
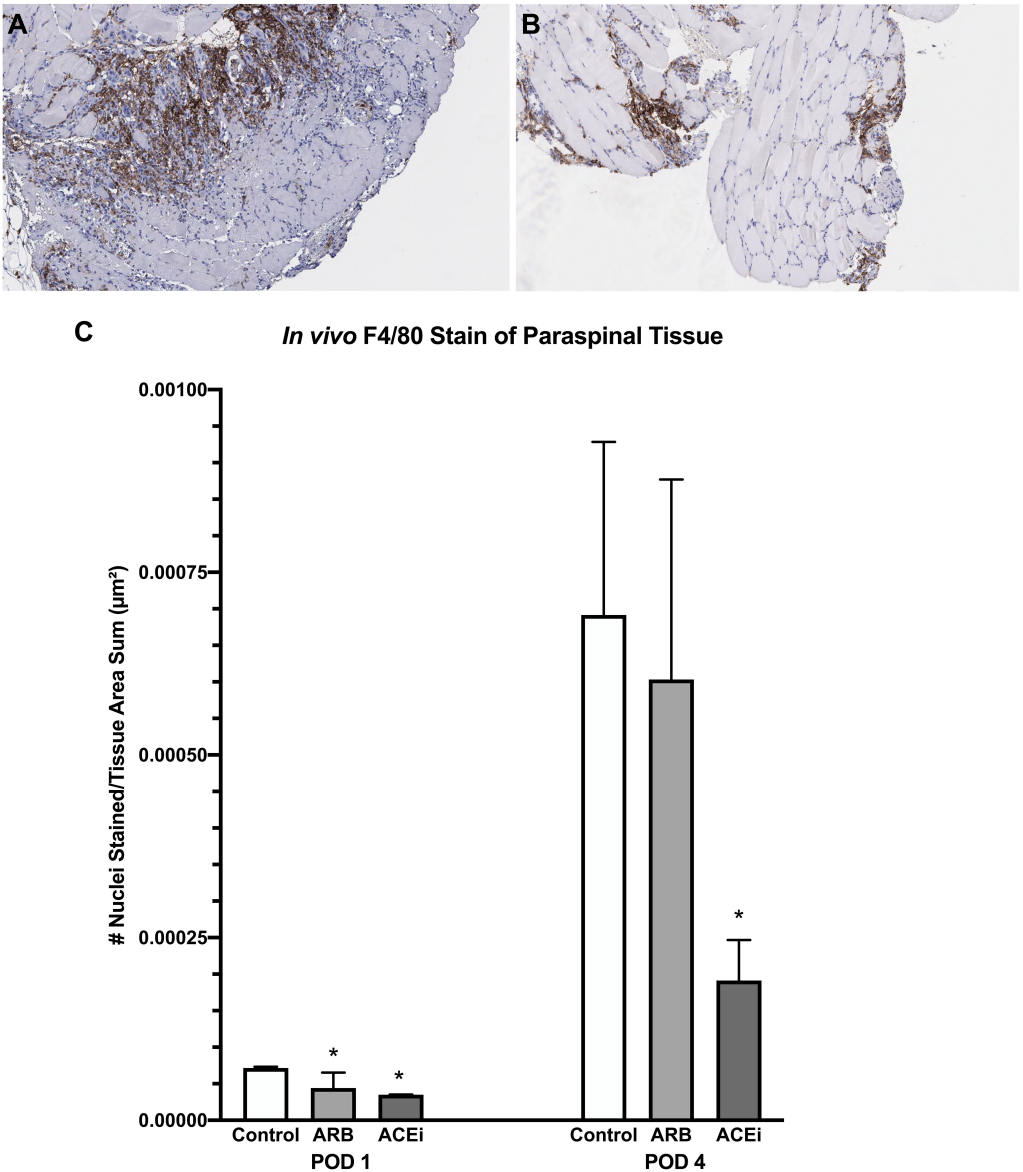
F4/80 stain representing monocyte infiltration to the paraspinal tissue in mice treated with an ARB **(A)** and an ACEi **(B)**. **(C)** Mice treated with both an ARB and ACEi had significantly lower levels of monocyte infiltration than the control group at POD 1. Mice treated with an ACEi had significantly lower monocyte infiltration than mice treated with an ARB and the control group at POD 4 **(C)**. ^*^p < 0.05.

**Figure 10:**
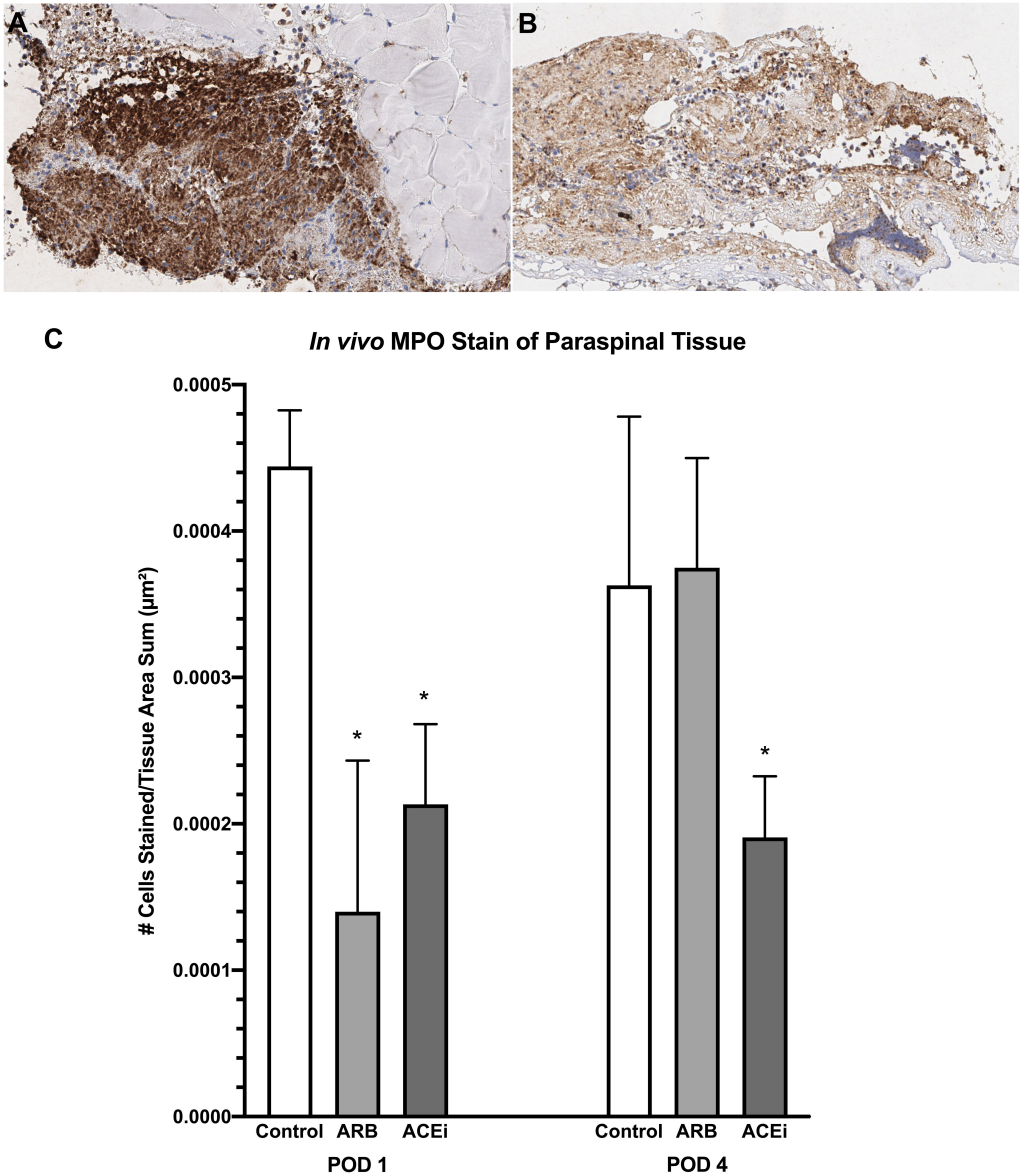
Myeloperoxidase stain representing neutrophil infiltration to the paraspinal tissue in mice treated with an ARB **(A)** and an ACEi **(B)**. **(C)** Mice treated with both an ARB and ACEi had significantly lower levels of neutrophil infiltration than the control group at POD 1. Mice treated with an ACEi had significantly lower neutrophil infiltration than mice treated with an ARB and the control group at POD 4 **(C)**. ^*^p < 0.05.

Following a review by a board-certified pathologist, there was no qualitative difference in monocyte or neutrophil infiltration to the spleen at POD1 or POD4 between the ACEi, ARB, or control treatment groups based on MPO and F4/80 stains. However, there was a qualitative difference in monocyte and neutrophil infiltration to the paraspinal tissue between the groups at POD 4 (Figures 9A,B, 10A,B). The number of nuclei stained at POD 4 per tissue area sum in samples stained with F4/80 was significantly lower in the ACEi group (1.9 × 10^−4^ ± 5.6 × 10^−5^) compared with both the ARB group (6.0 × 10^−4^ ± 2.7 × 10^−4^) and the infected control group (5.4 × 10^−4^ ± 3.9 × 10^−4^; *p* < 0.05; Figure 9C). The number of cells stained at POD 4 per tissue area sum in samples stained with MPO was significantly lower in the ACEi group vs. the ARB group (1.9 × 10^−4^ ± 4.2 × 10^−5^ vs. 3.8 × 10^−4^ ± 1.1 × 10^−4^, *p* < 0.05; Figure 10C).

## Discussion

Implant-associated SSI is a catastrophic complication. Moreover, staphylococcal species represent roughly two thirds of implant-associated SSI and often compound the issue due to multi-drug resistant phenotypes and a high propensity to form biofilms (65–67). Regardless of implant type, conservative, non-invasive treatment measures frequently fail to eradicate an infection and high-risk surgical intervention is often required (17, 19, 20). Thus, there is a significant and unmet need to identify modifiable risk factors that may optimize host immune protection against such infections. ACEi and ARB are amongst the most widely prescribed medications, particularly in the aging surgical population (31, 68). Given the prevalence of both ACEi and ARB, the potential impact of these therapies could be enormous if they modify immune response or efficacy in ways that subvert host defense.

The current findings first showed that neither ACEi nor ARB had any direct anti-staphylococcal activity *in vitro*. The rationale behind this *in vitro* experiment was to show that any purported impact of ACEi or ARB impact on bacterial burden *ex vivo* or *in vivo* would not have been due to any anti-staphylococcal effects of the actual therapeutics themselves. This study also shows that blood from mice treated with ACEi demonstrated significantly decreased respiratory burst capacity as well as a significantly decreased ability to suppress *S. aureus* infection *ex vivo* as compared to ARB-treated or control mice blood. Mice treated with an ACEi also had higher *S. aureus* burden *in vivo* as measured by bioluminescent signal throughout the entirety of the experiment compared to ARB-treated or infected control groups.

Congruent with these findings, CFU burden measured on implants as well as paraspinal tissue in the ACEi group was significantly higher than either the ARB or infected control groups. Furthermore, viable *S. aureus* CFUs were identified in 37.5% of the implants in the ACEi group as compared to 25% in both the ARB and infected control group. Paralleling these microbiologic findings, mice treated with ACEi had significantly decreased monocyte and neutrophil infiltration to the paraspinal tissue on POD 4 compared with infected controls or ARB-treated mice. These data suggest that monocyte and neutrophil infiltration and/or functional ROS generation might be impaired by ACEi-related mechanism(s) and may be responsible for the increased susceptibility to *S. aureus*. Taken together, these findings suggest that perioperative ACEi treatment may represent a previously unappreciated risk factor to be considered prior to high-risk surgery such as implant instrumentation.

The increased infectious burden that developed with ACEi treatment is consistent with emerging literature that the renin-angiotensin system, and in particular ACE-1, may play an important role in innate pathogen defense (34). Khan et al. (26) recently found that selective neutrophil underexpression of ACE-1 markedly increased the susceptibility of mice to cutaneous methicillin resistant *S. aureus* (MRSA) infection, whereas neutrophil ACE-1 overexpression reduced susceptibility. Similar results have been reported with selective ACE-1 expression modulation in macrophages when challenged with both MRSA and *Listeria monocytogenes* (40). In both of these studies, the effect of ACE-1 to enhance pathogen clearance appeared angiotensin type-1 receptor independent, consistent with the majority of the results of the present study.

However, other studies implicate the angiotensin type-1 receptor in diverse leukocyte functions including neutrophil chemotaxis (69) as well as natural killer cell proliferation and chemotaxis (70). Activated neutrophils are also a source of angiotensin-II generation, which are produced both ACE-dependently and independently (71). In our study, ARB-treated mice treated with ACEi showed significantly higher *ex vivo* CFU counts and significantly lower neutrophil and monocyte infiltration on POD 1 as compared to the infected control group. Therefore, while ARBs may influence pathogen defense-related pathways, these effects appear far less pronounced than ACE1 inhibition. Whether this distinction has clinical relevance warrants future investigation.

There are limitations to this study. It is important to consider the clinical, translational limitations of this implant infection model as it is a simplification of the complex steps involved in human spinal implant surgery. Limitations to this model include being unilateral, involving only the posterior elements of the spine, and use of a single stainless-steel metal implant (53). Furthermore, only *S. aureus* Xen36 was used in this study. Although this has been shown to be a well-validated, representative strain from a clinical isolate (57–59) the authors cannot extrapolate the findings reported to different staphylococcal strains or other microbial organisms. Given that this model allows for a safe, feasible, well-powered, and reproducible way to longitudinally quantify infection *in vivo*, these advantages are widely viewed to outweigh the accepted limitations. Another limitation to this study is the documented differences between murine and human physiology (38, 72, 73). Although doses of study therapeutics have been well-established and verified (40, 61), dose equivalent adjustments to humans as well as murine-specific pharmacological properties of these therapeutics are further limitations. However, mice treated with ACEi have been shown to respond similarly to humans in that they develop hypotension, increased levels of angiotensin I and decreased levels of ACE expression in myeloid cells (34, 38). Lastly, although this study showed that neither ACEi nor ARB exerted any direct antimicrobial effects on the growth potential of *S. aureus in vitro*, the potential direct effects of these study therapeutics on *S. aureus* metabolism, gene expression and/or virulence *in vivo* could also contribute to differences in outcomes observed.

The current findings provide *ex vivo* and *in vivo* evidence that perioperative ACEi treatment as compared to ARB treatment increases *S. aureus* burden in a manner that corresponds to a reduction in immune effector responses in a longitudinal murine implant infection model. These results in conjunction with the overall body of literature on ACEi immunomodulation suggest that perioperative ACEi treatment could pose additional infectious risks to patients. It is, however, important to consider the balance between any purported immunomodulatory effects of ACEi and its protective cardiovascular effects. ACE inhibition has been shown to improve arterial compliance (74, 75) and, by inhibiting angiotensin II formation, decrease left ventricular hypertrophy, generalized coagulability and possibly systemic sympathetic activity in diabetic and hypertensive patients (76–78). Therefore, the discontinuation of ACEi perioperatively is not without cardiologic risk. In patients lacking specific indications for particular antihypertensives, ACEi and ARB are often both considered first line therapy (79). Fortunately, ARB have been shown to exert protective cardiovascular effects to a similar, and perhaps greater, extent than ACEi (76, 78, 80, 81). Thus, the cardiovascular sequelae of switching a patient from an ACEi to an ARB perioperatively may not be substantially different. Moreover, unlike well-established modifiable host risk factors such as obesity and diabetes, switching a patient from ACEi to ARB treatment may be relatively easy, safe, and inexpensive. To this end, it may be possible that certain patients undergoing elective surgery could safely be switched from an ACEi to an ARB during the perioperative period to minimize any purported infectious risk associated with the immunomodulatory effects of ACEi treatment.

Preoperative host optimization is a key component to mitigating the risk of SSI and its devastating sequelae. The results of this study add to the growing body of literature suggesting that ACEi treatment may represent an under-appreciated, modifiable infectious risk factor. Future clinical studies investigating the relation between SSI and choice of antihypertensives are warranted to help develop guidelines regarding the perioperative use of ACEi.

## Data Availability Statement

The raw data supporting the conclusions of this article will be made available by the authors, without undue reservation.

## Ethics Statement

The animal study was reviewed and approved by ARC (Animal Research Committee) at the University of California, Los Angeles (UCLA).

## Author Contributions

RT, DG, BK, ZM, TS, NC, MY, and NB contributed to conception and design of the study. All experiments were performed by RT, DG, BK, ZM, TS, TO, AC, CM, DL, NC, AS, and NB. Mouse surgical procedures were performed by RT with assistance from DG, BK, ZM, TS, TO, AC, NC, and NB. NC performed the statistical analysis. RT, DG, TS, NC, and DL completed all reference formatting. Figure generation was done by RT, DG, BK, ZM, CM, and NB. RT, DG, BK, ZM, TS, NC, TO, AC, CM, DL, AS, MY, and NB all assisted in writing the first and all subsequent drafts of the manuscript. All authors read and approved the submitted manuscript.

## Conflict of Interest

NB and MY have or may receive grant support or related benefits from the National Institutes of Health. MY receives grant support from the U.S. Department of Defense, and is founder and shareholder of NovaDigm Therapeutics, Inc., which develops novel vaccines and immunotherapeutics targeting infection. The remaining authors declare that the research was conducted in the absence of any commercial or financial relationships that could be construed as a potential conflict of interest.
